# Chloroquine-Induced Accumulation of Autophagosomes and Lipids in the Endothelium

**DOI:** 10.3390/ijms22052401

**Published:** 2021-02-27

**Authors:** Ewelina Bik, Lukasz Mateuszuk, Jagoda Orleanska, Malgorzata Baranska, Stefan Chlopicki, Katarzyna Majzner

**Affiliations:** 1 Jagiellonian Centre for Experimental Therapeutics (JCET), Jagiellonian University, 14 Bobrzynskiego Str., 30-348 Krakow, Poland; ewelina.bik@doctral.edu.uj.edu.pl (E.B.); lukasz.mateuszuk@jcet.eu (L.M.); jagoda.orleanska@doctoral.uj.edu.pl (J.O.); m.baranska@uj.edu.pl (M.B.); stefan.chlopicki@jcet.eu (S.C.); 2 Faculty of Chemistry, Jagiellonian University, 2 Gronostajowa Str., 30-387 Krakow, Poland; 3 Chair of Pharmacology, Jagiellonian University, 16 Grzegorzecka Str., 31-531 Krakow, Poland

**Keywords:** endothelium, Chloroquine, lipids, lysosomotropism, Raman imaging, fluorescence imaging

## Abstract

Chloroquine (CQ) is an antimalarial drug known to inhibit autophagy flux by impairing autophagosome–lysosome fusion. We hypothesized that autophagy flux altered by CQ has a considerable influence on the lipid composition of endothelial cells. Thus, we investigated endothelial responses induced by CQ on human microvascular endothelial cells (HMEC-1). HMEC-1 cells after CQ exposure were measured using a combined methodology based on label-free Raman and fluorescence imaging. Raman spectroscopy was applied to characterize subtle chemical changes in lipid contents and their distribution in the cells, while the fluorescence staining (LipidTox, LysoTracker and LC3) was used as a reference method. The results showed that CQ was not toxic to endothelial cells and did not result in the endothelial inflammation at concentrations of 1–30 µM. Notwithstanding, it yielded an increased intensity of LipidTox, LysoTracker, and LC3 staining, suggesting changes in the content of neutral lipids, lysosomotropism, and autophagy inhibition, respectively. The CQ-induced endothelial response was associated with lipid accumulation and was characterized by Raman spectroscopy. CQ-induced autophagosome accumulation in the endothelium is featured by a pronounced alteration in the lipid profile, but not in the endothelial inflammation. Raman-based assessment of CQ-induced biochemical changes offers a better understanding of the autophagy mechanism in the endothelial cells.

## 1. Introduction

Chloroquine (CQ, an antimalarial drug with anti-inflammatory properties) is a well-known lysosomotropic compound. Chloroquine (CQ) and its derivatives came into the spotlight of the clinical interest in the context of the ongoing COVID-19 pandemic [1,2,3]. By chemical nature, CQ belongs to cationic amphiphilic drugs (CADs); it is a weak base due to its chemical properties (partition coefficient clogP = 4.63, and the negative logarithm of the dissociation constant, pKa, is equal to 10.1). CQ easily goes through membranous compartments and accumulates in acidic organelles where it is protonated and trapped, yielding an increase in the pH of this environment. Lysosomes are regulators of lipid metabolism [4], and intact autophagy is required for the maintenance of lipid homeostasis in the endothelium [5]. CQ with its inhibitory effects on lysosomes and autophagy [6,7] has a considerable influence on lipid metabolism, emerging as a key regulator of inflammation [8,9], proliferation [10], hypoxia [11], fat buffering [12], stress responses [13], and also endothelial cell death [14]. Pathological states, which result from endothelium perturbations, are mainly cardiovascular and metabolic complications [15]. CQ inhibits autophagy, but knowledge of the autophagy modulation by CQ in the endothelium is limited [16]. Therefore, it is not known whether CQ, by influencing the lysosomes and/or autophagosomes, leads to abnormalities in the metabolism of lipids in the endothelium. CADs are thought to alter the gene expression by hindering the activity of lysosomal phospholipases and enzyme transport but increasing the production of phospholipids, cholesterol, and even drugs or their metabolite accumulation. CQ as a member of CADs induces changes in cell metabolism and leads to phospholipidosis, defined as a lysosomal storage disorder [17] resulting in abnormal accumulation of phospholipids in tissues and lamellar body formation [18,19].

The aim of this study was to characterize the biochemical changes in the endothelial cells in response to CQ treatment. In particular, we determined the composition of lipids in cells after CQ treatment with the use of Raman imaging, a method of high sensitivity for lipid detection [20].

## 2. Results and Discussion

### 2.1. Characterization of Endothelial Responses to CQ

**Figure 1:**
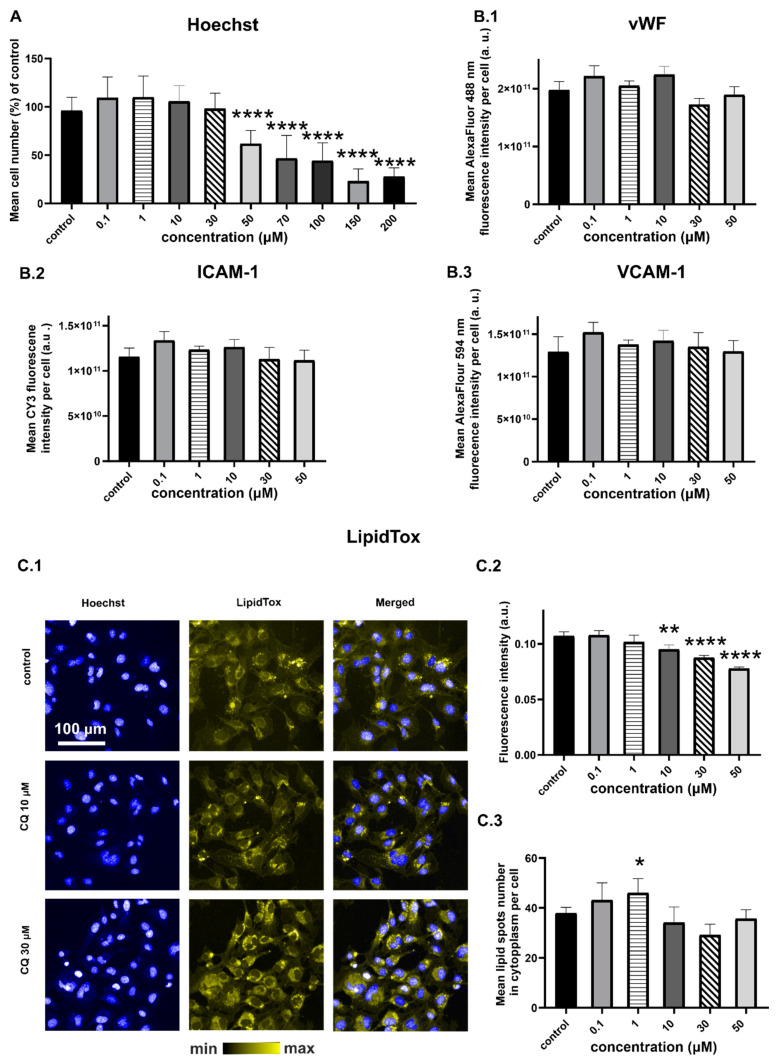
HMEC-1 response to 24-h incubation with CQ: (**A**) Number of cells calculated based on Hoechst staining and presented as a % of the control for the nucleus number; (**B.1**) vWF; (**B.2**) ICAM-1; (**B.3**) VCAM-1 expression (ANOVA; * *p*-value < 0.05, ** *p*-value < 0.01, **** *p*-value < 0.0001); representative fluorescence images of cells stained with Hoechst and LipidTox (**C.1**) fluorescence intensity of lipid spots (**C.2**) and their mean number in the cytoplasm (**C.3**). CQ affected cell viability at 50 µM, had no effect on vWF, VCAM-1, ICAM-1 overexpression, and increased the neutral lipid content (ANOVA, *p*-value < 0.05). Average fluorescence value from six wells was quantified from >9000 cells per well. Results were obtained from three independent experiments.

CQ treatment resulted in a concentration-dependent decrease in the number of endothelial cells (Figure 1A), indicating CQ toxicity at the concentrations of 50 µM and higher. However, the expression of intercellular adhesive (ICAM-1), vascular cell adhesion (VCAM-1), and von Willebrand factor (vWF) (Figure 1B) did not change in response to CQ across the whole range of used concentrations. Thus, reduced viability of cells (Figure 1A) was not the effect of inflammatory responses that were detected as an overexpression of ICAM-1, VCAM-1, or vWF. In turn, fluorescence staining for neutral lipids (LipidTox, Figure 1C) showed a statistically significant increase (ANOVA; *p* ≤ 0.05) in the number of cytoplasmic lipid spots in the cells treated with CQ, and the effect was most pronounced at the concentration of 30 µM.

### 2.2. Efects of CQ on Lysosomes, Phospholipidosis, and Autophagy

**Figure 2:**
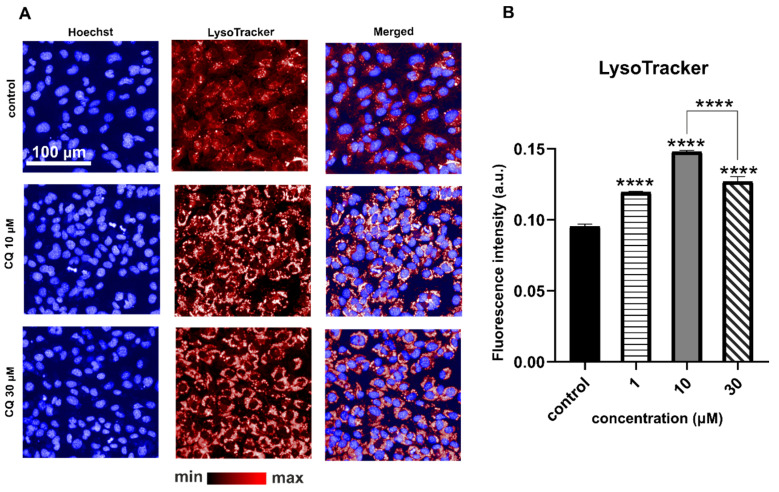
Representative fluorescence images of LysoTracker Deep Red staining of HMEC-1 for control and CQ-treated cells (**A**). Quantification of the fluorescence intensity (**B**); ANOVA **** *p*-value < 0.0001) for LysoTracker reveals significantly increased lysosomal volume for cells treated with 1, 10, 30 µM CQ in comparison to the untreated control. Average fluorescence value from three wells was quantified from >9000 cells per well. Results were obtained from three independent experiments. Color differences in the fluorescence image are related to the background noise and maximum values of the fluorescence intensity for control and CQ-treated cells.

LysoTracker-based fluorescent staining specific for lysosomal structures and other acidic organelles was applied to analyze the effects of CQ on lysosomes. Increased LysoTracker fluorescence intensity was observed for cells treated with CQ (1, 10, and 30 µM), as shown in Figure 2. It has been previously demonstrated that increased LysoTracker intensity in response to CADs was related to lysosomal biogenesis and/or increased lysosomal activity [16]. It is possible that treatment with highly alkaline lysosomotropic compounds increased the number of lysosomes in cells in response to increased cellular pH. However, in our study, LysoTracker intensity in cells treated with 30 µM CQ decreased in comparison to those exposed to 10 µM CQ. That difference is probably related to CQ accumulation, observed by means of Raman imaging, described below. CQ at a concentration of 30 µM increased the number of lysosomes, but then accumulated in their interior area, causing its neutralization and subsequently decreasing the fluorescence intensity of LysoTracker. A slight decrease in LysoTracker intensity at 30 µM CQ in comparison to 10 µM may also suggest that the Golgi apparatus could be disrupted, but to verify this issue directly, further studies are required.

**Figure 3:**
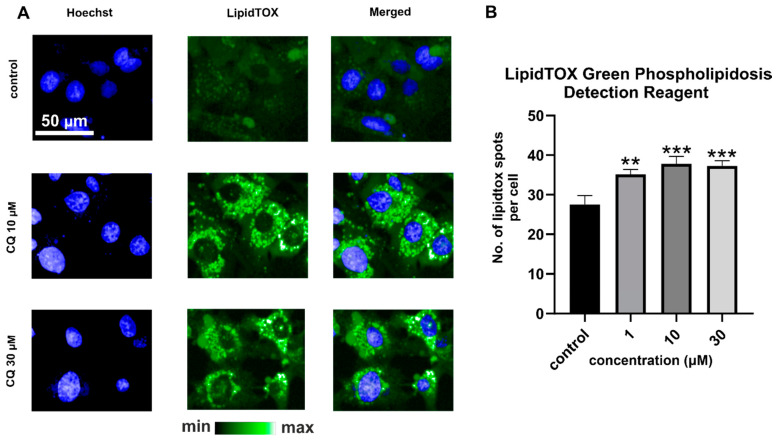
Representative fluorescence images of LipidTOX Phospholipids Green staining of HMEC-1 for control and CQ-treated cells (**A**). Quantification of the fluorescence intensity (**B**); ANOVA *** *p* value < 0.001, ** *p* value < 0.01) for LipidTOX Phospholipid Detection Reagent reveals significantly increased phospholipid contents for cells treated with 1, 10, and 30 μM CQ in comparison to untreated control. The average fluorescence value from three wells was quantified from >9000 cells per well. Results were obtained from three independent experiments.

In this paper, two types of HCS LipidTox staining were applied—LipidTox Deep Red Neutral Lipid Stain (Figure 1C) and LipidTOX Green Phospholipidosis Detection Reagent (Figure 3), both suitable to study toxic side effects of compounds on lipid metabolism in mammalian cell lines. According to the manufacturer, the LipidTox Deep Red has high specificity to neutral lipids and an extremely high affinity for neutral lipid droplets. The LipidTOX Green Phospholipidosis Detection Reagent is dedicated to phospholipidosis detection, which is often triggered by cationic amphiphilic drugs. Within 24 h of incubation with CQ, the increase in fluorescence intensity of LipidTOX for phospholipidosis detection was significant.

**Figure 4:**
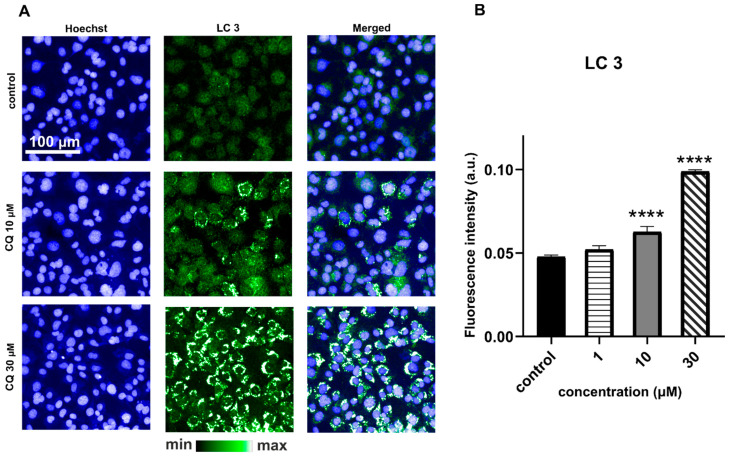
Immunofluorescence images of LC3 staining of HMEC-1 for control and CQ-treated cells (**A**). Quantification of the LC3 fluorescence intensity (**B**; ANOVA **** *p* < 0.0001) showed significantly increased autophagosome numbers for cells treated with 10 and 30 µM CQ in comparison to the untreated control. The average fluorescence value from three wells was quantified from >9000 cells per well. Results were obtained from four independent measurements in reference to the untreated control.

Autophagosomes, with pH around 6 [21], could also affect the fluorescent intensity of LysoTracker. In the context of CADs, this hypothesis was discussed before, since CQ is widely used as an autophagy inhibitor [6,22,23]. The acidic vesicular organelles could be a marker of autophagy, connected to the conversion of microtubule-associated protein 1 light chain (LC3); therefore, to confirm the hypothesis about the relation between CQ and autophagy, we performed immunocytochemistry for LC3 (Figure 4). As expected, the CQ-treated cells at 10 and 30 µM concentrations were characterized by much larger LC3-positive structures compared to the control cells. The lipidated form of LC3 (known as LC3-II) is usually linked to the mature stage of autophagosomes. The autophagosome formation results in LC3 cleavage into LC3-I, which may be later transformed into a lipidated form by conjugation to phosphatidylethanolamine (L3-II). The LC3-II incorporated into autophagosomes is a clear indicator of autophagy, which may lead to the late stage of autophagy, including autophagosome fusion with lysosomes and/or lysosomal degradation [24]. CQ resulted in late autophagic inhibition by impairing autophagosome–lysosome fusion, similar to bafilomycin A_1_ [25]. It was believed that those two compounds may block autophagy by a lysosomal pH increase, reducing the activity of lysosomal enzymes and their degradation capacity as a result. However, in [6], the authors claimed that autophagy inhibition by CQ indirectly comes from Golgi disorganization and the effect on endosomal function [6,23]. Therefore, increased autophagosome accumulation demonstrated in Figure 4 is consistent with previously reported results. At a lower CQ concentration (1 µM), fluorescence specific for LC3-possitive structures was not observed, but LysoTracker staining showed changes in the lysosomal and acidic structures of cells treated with 1 µM CQ (*p*-value < 0.0001).

### 2.3. Characterization of Lipid Changes Induced by CQ Using RAMAN Imaging

**Figure 5:**
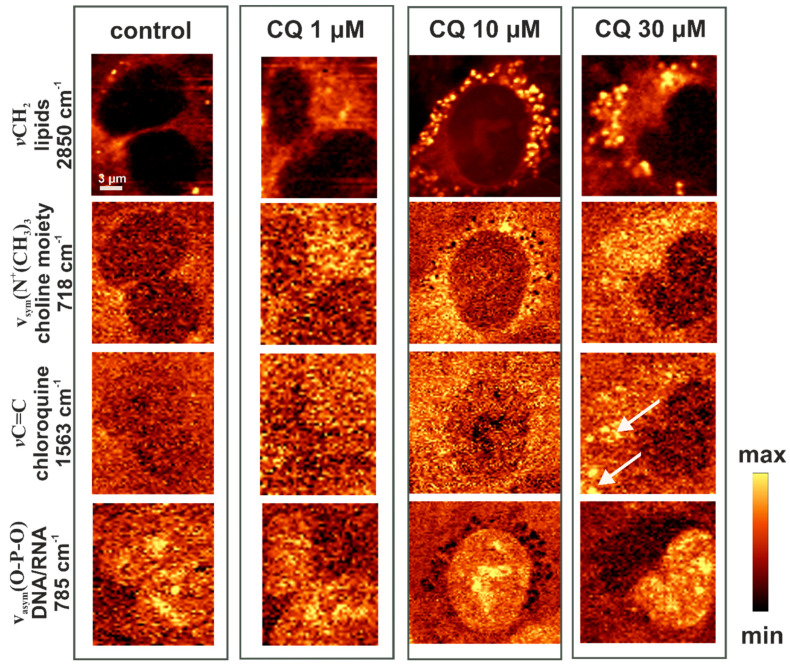
Examples of Raman images of the HMEC-1 cells: the control cells and cells incubated with 1, 10, and 30 µM of CQ for 24 h. Raman maps based on specific bands were obtained with a 532 nm laser wavelength and with a sampling density of 0.3 μm, which clearly shows the lipid accumulation in the CQ-treated cells. Arrows indicate CQ accumulations in cells.

To better understand the biochemical alterations observed with LysoTracker staining and LC3 immunocytofluorescence in HMEC-1 upon CQ treatment, Raman imaging was performed. HMEC-1 cells were treated with CQ under the same conditions as for fluorescence imaging (24 h at 1, 10, and 30 µM concentration). CQ treatment caused accumulation of lipids within the endoplasmic reticulum and perinuclear region. In Figure 5, Raman images show the distribution of selected biochemical components of the cells and CQ, based on the integral intensity of characteristic bands for each chemical species. Raman images showed cellular CQ at a concentration 30 µM, clearly indicating its lysosomotropic properties based on characteristic 1563 cm^−1^ band (as well as 1378 cm^−1^—data not shown here) assigned to *ν*C = C in quinoline and *ν*C = C in the *δ*CH quinolone of CQ [26]. The integration map of the band at 718 cm^−1^ for the choline moiety (*v_sym_* (N^+^ (CH_3_)_3_) shows the distribution of choline-containing lipids, such as phosphatidylcholine and sphingomyelin, the major phospholipids in eukaryotic cells [27]. Integration of the band at 2850 cm^−1^ reflects the overall lipid distribution and abundance (*v*CH_2_ in the range of 2830–2900 cm^−1^). The nucleus was visualized by integration of a band characteristic of nucleic acids (*v_asym_*(O-P-O) at 785 cm^−1^). Regions in Raman images with an increased content of lipids and choline-containing lipids after stimulation with CQ show similar distribution in the perinuclear region, such as LysoTracker, LC3, and LipidTox (Figure 1, Figure 2, Figure 3 and Figure 4). These results clearly showed that CQ, a well-known lysosomotropic drug, induced an increase in the cellular lipid content.

The number of spots related to lipids in general and choline-containing lipids in particular, as well as their Raman intensity, was noticeably higher after the treatment with 10 and 30 µM of CQ (Figure 5), which was confirmed by the increased fluorescence intensity of LipidTox Phospholipid Green staining (Figure 3). Since, after 24 h of CQ-treatment a pronounced increase in the size of lipid spots and their fluorescence intensity was observed, increased lysosomal volume can be hypothesized.

**Figure 6:**
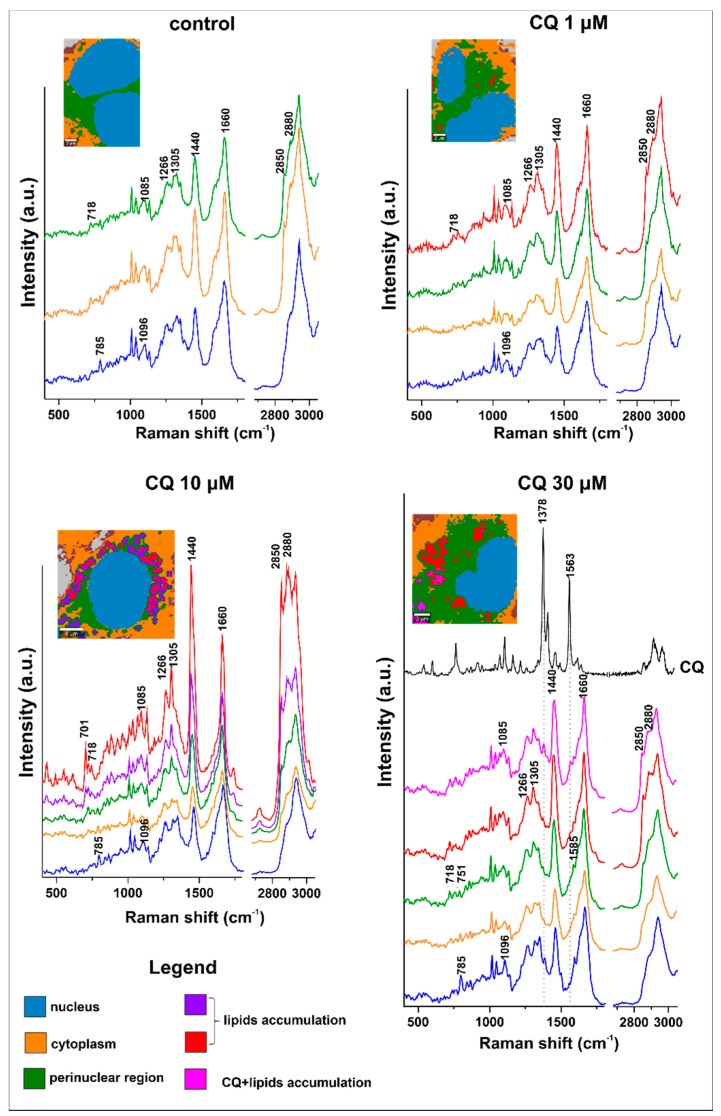
Representative KMCA of the lysosomotropic effect of CQ (30 μM) on HMEC-1. Average spectra of CQ with lipids, lipids, perinuclear region, cytoplasm, and nucleus were generated (color-coded with the KMCA image) based on KMCA and the reference spectrum of CQ in the solid-state.

The average spectra of newly formed lipid accumulations were obtained by k-means cluster analysis (KMCA) for which further analysis and comparison were conducted. This approach enabled us to extract average spectra from cell organelles that vary in biochemical composition, enabling us to reduce the very high number of single spectra and to visualize the cell classes. Representative results of KMCA are presented in Figure 6 as a general idea of this analysis. Such an approach allowed discrimination of a newly formed class of lipid accumulations (marked in red and violet) after CQ treatment, based on increased intensities of the typical Raman signature of lipids: 1305 cm^−1^ (*τ*CH_2_/CH_3_), 1440 cm^−1^ (*δ*CH_2_), 1660 cm^−1^ (*v*C = C), 2850 and 2880 cm^−1^ due to *τ*CH_2_/CH_3_. A nucleus class (marked in blue) was separated based on typical marker bands of nucleic acids at 785 and 1096 cm^−1^ due to *v_asym_*(O-P-O) and *v*(PO_2−_), respectively. The perinuclear area class (marked in green), rich in membranous organelles such as the endoplasmic reticulum, Golgi apparatus, lysosomes, and mitochondria, was separated from the cytoplasm (orange) by an overall higher Raman signal resulting from a higher contribution of cytochromes (751, 1128, 1315, and 1585 cm^−1^), proteins which are present in mitochondria and higher amounts of lipids (2850 cm^−1^), as well as choline-containing lipids manifested by a higher intensity of bands at 718 (*v*[-N^+^(CH_3_)_3_]) and 1089 cm^−1^ *v*(PO_4_^3−^) [28,29]. The magenta class is a mixture of lipids and CQ; it has a typical lipidic spectral profile and two characteristic CQ bands such as 1378 and 1563 cm^−1^.

**Figure 7:**
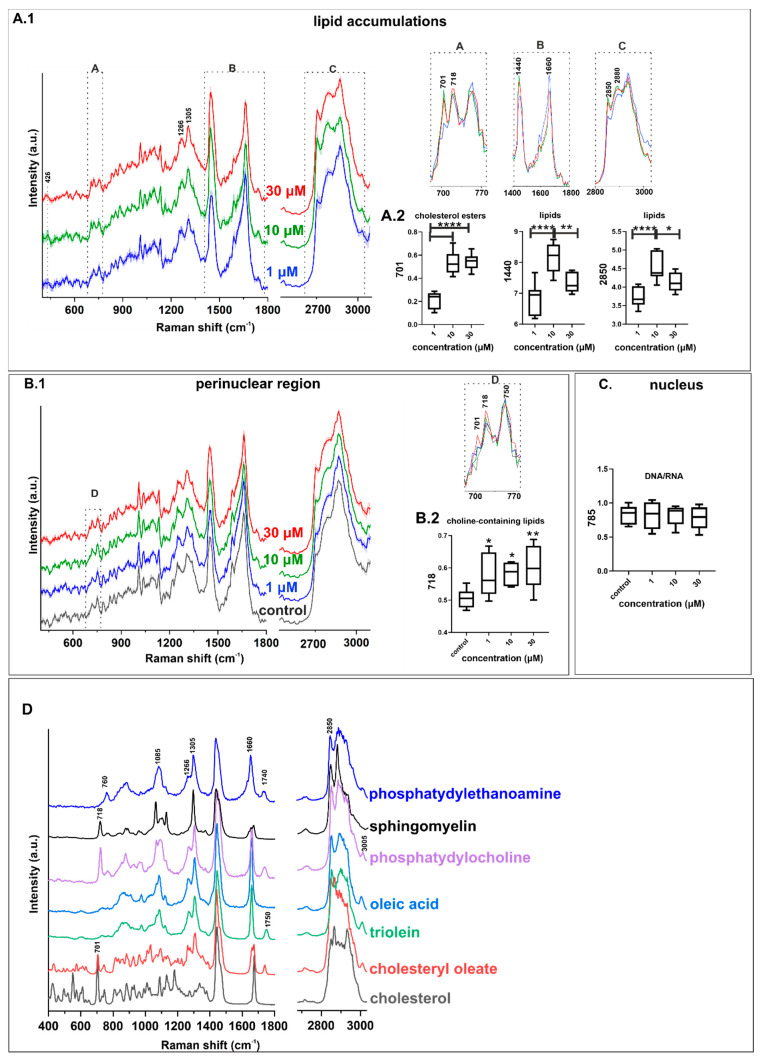
Average Raman spectra (±SD) of accumulated lipids (**A.1**) with the integral intensities of cholesterol esters (701 cm^−1^) and overall lipids (1440 and 2850 cm^−1^) (**A.2**), (**B.1**) Averaged Raman spectra (±SD) of the perinuclear region with the integral intensities of choline-containing lipids marker band (718 cm^−1^) (**B.2**,**C**) integral intensity of nucleic acids (785 cm^−1^) marker band in the nuclear area for HMEC-1 treated with 1, 10, 30 µM of CQ. (ANOVA; * *p* < 0.05, ** *p* < 0.01, **** *p* < 0.0001; *n* = 10 per treatment condition, three independent experiments). (**D**) Reference Raman spectra of lipids, obtained with 532 nm laser line.

Average spectra of lipid accumulations identified in cells treated with CQ are presented in Figure 7. A direct comparison of spectra from 1, 10, and 30 µM of CQ-treated cells indicates increased intensities of the typical Raman signature of lipids (described above) and choline-containing lipids (718 cm^−1^). Intensities of those Raman features were significantly higher for cells treated with 10 and 30 µM of CQ than with 1 µM, which was determined by quantitative analysis based on the band integral intensities (ANOVA, *** *p* < 0.001).

Figure 5 shows an increased content and distribution of lipids in comparison to control cells, as described above. Spectra presented in Figure 7 were characterized by protein-specific signal traces (1004 cm^−1^, Phe). The appearance of proteins in a class of lipid deposits can be expected due to the presence of specific proteins associated with lysosomes and autophagosomes, which are composed of soluble and transmembrane proteins found on their surfaces [30,31]. In the present study, for semi-quantitative analysis, imaging was performed with density probing of 1 µm, which affected the image resolution but increased the number of cells measured in a reasonable time.

Even though some general similarities in the lipid profiles observed in the Raman images of lipid deposits were found, their composition varied between used concentrations of CQ. The main differences are related to the presence of 426, 701, and 741 cm^−1^ (characteristic bands for cholesterol [32]) and 1750 cm^−1^ (C=O stretching band indicates esterified form) bands, which indicate the presence of cholesterol esters [32] in lipid accumulations, detected after incubation with CQ 10 and 30 µM. Moreover, the spectra of lipids found in cells treated with a higher CQ concentration manifested a typical spectral profile of unsaturated lipids by the presence of 1266 cm^−1^ (*δ* = CH), 1660 (*v*C = C) cm^−1^, and low intensity of 3005 cm^−1^ Raman features. The spectral shape and ratio of 1266/1305 or 1660/1440 cm^−1^ suggested monounsaturated lipids in the composition [32,33]. For lower CQ concentrations, the protein content affected the spectroscopic signal from the class of lipid accumulations. Protein traces in the lipid deposits in cells treated with 1 µM CQ can be related to biological and technical aspects. The appearance of proteins in lipidic structures might be expected due to the presence of specific proteins associated with their structures as observed for lipid droplets. CQ treatment increased the content of choline-containing lipids in the perinuclear region (Figure 7B) but did not affect the nucleic acid signal intensity from the nuclear class as shown in Figure 7C. Raman imaging does not provide an adequate spatial resolution to investigate proteins exclusively attached to cellular or subcellular membranes, since the recorded Raman signal must be averaged from the whole volume of spots. Raman intensities of lipid accumulations and associated proteins can be related to protein aggregation around those structures. The technical aspect of mixed signals from lipids and proteins is related to the lateral resolution of the Raman system, applied sampling density, and finally, the voxel size that can also affect the average signal collected not only from the focal plane of lipid accumulations but also from the area above and/or below.

## 3. Materials and Methods

### 3.1. Cell Culture

Human dermal microvascular endothelial cells (HMEC-1) were maintained in complete MCDB131 medium (Gibco, Life Technologies, Grand Island, NY, USA) at 37 °C/5% CO_2_. HMEC-1 (3rd passage) cells were seeded at a concentration of 18 × 104 and left for 24 h on CaF2 slides (Crystran LTD, Poole, Dorset, UK). For Raman measurements, cells were treated with various concentrations of CQ for 24 h. Then cells were fixed using 2.5% solution of glutaraldehyde in phosphate-buffered saline (PBS; Gibco, Life Technologies, Grand Island, NY, USA) for 5 min. The cell density seeded for fluorescence imaging was 3 × 104 per well.

### 3.2. Raman Measurements with Data Analysis and Processing

Raman images were acquired using a confocal Raman microscope (WITec alpha300, Ulm, Germany) supplied with air-cooled solid-state lasers operating at 532 nm. A water immersion objective (60 × 1.0 NA) was applied, and the power of the laser at the sample position was ca. 30 mW. Data were collected with an acquisition time per spectrum of 0.5 s, sampling density of 1 μm, and a spectral resolution of 3 cm^−1^. For each of three independent experiments, ten cells were measured per treatment condition. K-means cluster analysis (KMCA) was performed to define major cellular structures (nucleus, perinuclear area (lipid-rich cytoplasmic area with a strong contribution of endoplasmic reticulum), lipid accumulation, and protein-rich cytoplasmic area, of which average spectra were further analyzed and provided information about the biochemical changes between the control and CQ-treated cells. Data acquisition was controlled by the WITec alpha 300 software package. Pre-processing included cosmic spike removal, and background subtraction (using a polynomial fit, order 3) was performed using Project FIVE 5.1 Plus software (WITec GmbH, Ulm, Germany). Raman images were generated by using a summed filter -signal intensity over a defined wavenumber range representative of the molecular vibrations of interest, which were integrated and the background subtracted as a linear baseline from the first to second border as defined by the summed filter.

### 3.3. Fluorescent and Immunofluorescent Staining

HMEC-1 cells were seeded in 96-well plates and left overnight to attach. After CQ treatment, cells were stained with the LysoTracker Deep Red probe (Invitrogen, Carlsbad, CA, USA) and Hoechst 33342 (Invitrogen, Carlsbad, CA, USA) in triplicate wells. LysoTracker Deep Red was diluted to a 60 nM concentration and applied in a 1-h incubation, after which cells were stained with Hoechst 33342 (1:1000). The measurement was performed on live cells in cell culture media without phenol red, using a CQ1 automated confocal microscope (Yokogawa, Tokyo, Japan) with an excitation wavelength of 647 nm and an emission wavelength of 668 nm for Lysotracker Deep Red and wavelengths of 350 nm and 461 nm, respectively, for Hoechst. Side effects of CQ on lipid metabolism were detected with LipidTox Green Phospholipidosis Detection Reagent (Invitrogen, Carlsbad, CA, USA). LipidTox was diluted in full media (1:500) and then filtered with a syringe filter (2 µm pore size). Cells were loaded with LipidTOX simultaneously with the drug treatment according to the manufacturer’s protocol. Then, cells were fixed and counterstained with Hoechst (1:1000) for 10 min. The excitation/emission values of LipidTox were 495/525 nm.

The expression of VCAM-1, ICAM-1, and vWF was assessed using immunofluorescent staining (six wells per CQ concentration). After 24-h exposure to CQ, cells were fixed with 4% buffered formalin for 10 min. Primary antibodies were added for 24 h after initial permeabilization and the blocking step. The following primary antibodies were used: rabbit-anti-mouse von Willebrand factor (Abcam, Cambridge, UK; 1:100), mouse ICAM-1 (ThermoFisher, Waltham, MA, USA; 1:250), and rat VCAM-1 (ThermoFisher, Waltham, MA, USA; 1:200). After overnight incubation, cells were washed twice in PBS before the addition of secondary antibodies (all from Jackson ImmunoResearch Europe Ltd, Cambridge, UK); Alexa Fluor 488-conjugated goat-anti-rabbit (excitation: 493 nm, emission: 519 nm), Cy3-conjugated goat-anti-rat (excitation: 550 nm, emission: 570 nm) or Biot SP-conjugated goat-anti-rat, followed by Alexa Fluor 594-conjugated streptavidin (excitation: 591 nm, emission: 614 nm) and nuclear counterstaining (Hoechst 33258), enabling evaluation of the cytotoxicity based on cell count. Lipids were visualized by LipidTox Deep Red Neutral Lipid staining (Invitrogen, Carlsbad, CA, USA; 1:200).

For LC3 assessment, cells were treated with CQ for 24 h in triplicate wells. The LC3 Detection Kit (ThermoFisher, Invitrogen, Carlsbad, CA, USA) was employed to stain LC3 based on the manufacturer’s protocol. Cells were fixed with 4% buffered formalin for 10 min. After permeabilization with Triton, cells were incubated with primary antibody in normal serum goat solution for 1 h at room temperature. After washing three times in PBS, cells were incubated with secondary antibody (Alexa Fluor 488 goat-anti-rabbit) and Hoechst 33258 for nucleus counterstaining. Each staining was performed in a minimum of three independent experiments. All fluorescent images were captured with CQ1 Yokogawa, an automated, confocal fluorescence microscope, using a 20x objective. For each well, six fields with ~1500 cells per field (six planes along the z-axis from one field) were measured.

The fluorescence intensity of LipidTox, LysoTracker, and LC3 was assessed by using a spot intensity algorithm (Columbus software, Perkin Elmer, Waltham, MA, USA), while the expression of VCAM-1, ICAM-1, and vWF, as well as the intensity of the cytoplasm, was calculated per number of cells.

## 4. Conclusions

Changes in the chemical composition of endothelial cells in response to CQ are featured by lipid accumulations within the perinuclear region and cytoplasm. The lipid accumulations were characterized by in vitro Raman and fluorescent analyses of HMEC-1 cells treated with CQ at a micromolar range of concentrations. Spectral profiles of lipid deposits strongly depended on the CQ concentration. However, there was no linear relationship between the total amount of cellular lipids and the concentration of the drug. While choline-containing lipid signals increased for all cells treated with CQ, Raman features of cholesterol esters (characterised by bands at 426, 701, 1750 cm^−1^) and an increased total lipid content (a band at 2850 cm^−1^) were observed in the endothelial cells treated with the higher CQ (10 and 30 µM) concentrations. The important observation of this study was the demonstration that endothelial cells treated with CQ showed an increased content of lipids with the choline moiety (718 cm^−1^) in the perinuclear region. Fluorescence and immunofluorescence (LysoTracker and LC3) staining confirmed the accumulation of lysosomes and autophagosomes in the endothelial cell exposed to CQ, suggesting that the occurrence of lipid deposits was related to lysosomotropism and autophagy inhibition by CQ. Further studies are needed to understand better the link between changes in lipid composition and accumulation in the cytoplasm and the perinuclear region with the mechanism of autophagy in the endothelial cells.

## Data Availability

The datasets generated during and/or analysed during the current study are not publicly available due their massive file size but are available from the corresponding author on reasonable request.
